# Skills required by bachelor nursing students to meet the challenges of digitalisation in healthcare – results of qualitative expert interviews

**DOI:** 10.3205/zma001758

**Published:** 2025-06-16

**Authors:** Sarah Palmdorf, Annika Behler, Karina Ilskens, Christa Büker, Änne-Dörte Latteck

**Affiliations:** 1 Hochschule Bielefeld, Institut für Bildungs- und Versorgungsforschung im Gesundheitsbereich, Bielefeld, Germany; 2 Franziskus-Hospital-Haderberg, Stabsstelle Pflegewissenschaft, Georgsmarienhütte, Germany; 3 Das Fachworthaus – Lektorat Behler, Bielefeld, Germany

**Keywords:** nursing education, bachelor degree course, digitalisation, digital skills

## Abstract

**Objective:**

Through the digital transformation the everyday working life of nursing staff and the demands made on them are steadily changing. To meet these demands, Bachelor students need to learn appropriate “digital” skills during their courses. This study aimed at developing a module that imparts these digital skills. It was not clear which concrete skills the students should acquire and which topics should therefore be dealt with in the module.

**Methods:**

24 semi-structured guide-based interviews with experts were carried out and analyzed with qualitative content analysis. The experts came from the field of science, technology manufacturing, practical applications/practitioners and pedagogics.

**Results:**

We identified 4 different areas of skills: (1) organizing successful collaboration with patients, relatives and other professionals (doctors, therapists, IT); (2) supporting patients and relatives in the use of assistive technologies; (3) planning and implementing projects related to assistive technologies (4) managing lifelong learning in relation to assistive technologies. A reflective use of digital technologies in patient care and the education of patients and relatives were described as key qualifications.

**Conclusions:**

On the basis of the different assessments of the experts, it was possible to identify and discuss a wide range of digital skills. This can be used to define learning objectives for appropriate modules or to evaluate existing ones. The study therefore contributes to evidence-based teaching.

## Zusammenfassung

**Zielsetzung:**

Durch die digitale Transformation verändern sich der Arbeitsalltag und die Anforderungen an Pflegefachpersonen stetig. Um diesen Anforderungen gerecht zu werden, müssen Studierende während ihres Pflege-Bachelorstudiums entsprechende „digitale“ Fähigkeiten erlernen. Ziel dieser Studie war es, ein Modul zu entwickeln, das diese digitalen Fähigkeiten vermittelt. Dabei war es unklar, welche konkreten Fähigkeiten die Studierenden erwerben sollten und welche Themen daher im Modul behandelt werden sollten.

**Methoden:**

Es wurden 24 halbstrukturierte leitfadengestützte Expert:inneninterviews durchgeführt und mit qualitativer Inhaltsanalyse ausgewertet. Die Expert:innen kamen aus den Bereichen Wissenschaft, Technologieentwicklung, Pflege und Pädagogik.

**Ergebnisse:**

Es wurden vier verschiedene Kompetenzbereiche identifiziert: (1) Gestaltung einer erfolgreichen Zusammenarbeit mit Patient:innen, Angehörigen und anderen Berufsgruppen (Ärzt:innen, Therapeut:innen, IT); (2) Unterstützung von Patient:innen und Angehörigen bei der Nutzung von assistiven Technologien; (3) Planung und Durchführung von Projektvorhaben im Zusammenhang mit assistiven Technologien; (4) lebenslanges Lernen in Bezug auf assistive Technologien gestalten. Die reflektierte Anwendung digitaler Technologien in der Versorgung und die Beratung von Patient:innen und Angehörigen wurden als zentrale Fähigkeiten identifiziert.

**Schlussfolgerungen:**

Auf der Grundlage der unterschiedlichen Einschätzungen der Expert:innen konnte eine Vielzahl digitaler Fähigkeiten eruiert und diskutiert werden. Diese können genutzt werden, um Lernziele für entsprechende Module zu definieren oder bestehende zu evaluieren. Die Studie leistet damit einen Beitrag zur evidenzbasierten Lehre.

## 1. Introduction

The digital transformation of healthcare systems and institutions changes the everyday working life of nurses [1], [2], [3]. On the one hand, digitisation encompasses digital documentation within care structures [4], [5]. This is also accompanied by the digital communication of information with other professional players, possibly a stronger acceleration of process flows within organizations, e.g., via digital to-do lists, or the change in the type of documentation of patient data, e.g., in the form of stronger structuring through defined text modules [6], [7]. Digitisation in the area of communication also includes the use of digital media, e.g., for the education of patients and their families or for the continuing education of nurses. Another aspect of the changes brought about by digitisation involves the use of assistive technologies to support care processes. Assistive technologies can be defined as technological devices aimed “(...) to maintain or improve an individual’s functioning and independence to facilitate participation and to enhance overall well-being” [8]. The variance of technologies is very broad, ranging from less complex technology such as GPS tracking to AI-driven technology that detects care needs based on behavioural patterns [9], [10], [11]. 

Depending on the patient group, other skills and a deeper level of knowledge are also essential in order to provide effective care. For example, in the case of people with cognitive impairments such as dementia, it is important to understand the specific needs of patients and relatives with regard to technology-assisted care in order to counteract care problems [12]. The exploration of these issues creates a deeper understanding of ethical challenges, reciprocal needs relationships between the patient and his or her environment, and the anticipatory addressing of future care problems.

To ensure contemporary, high-quality technology-assisted healthcare, skilled nurses are required. They need to have digital, reflective and science-based skills in addition to clinical, pedagogical and administrative skills in order to fulfill an effective role in a constantly changing healthcare system. In order to cope with these changes, nurses need specific knowledge and skills [13], [14], [15], [16]. Previous nursing courses in Germany have focused on the topic of nursing informatics. It is therefore essential to discuss the skills required beyond this. 

Internationally, there are several studies that describe skills related to the use of assistive technologies. Different skills are discussed:

1. education of patients and relatives,
2. technical skills in the use of new technologies, e.g. to maintain care at a distance,
3. coordination, communication and participation with all stakeholders,
4. soft skills (new communication skills, adaptability and problem solving skills),
5. self-development and information about new technical solutions,
6. handling of data and media and
7. participation in the development and introduction of technologies in care [15], [17], [18].

However, comparability is limited due to differences in focus. A national perspective is also necessary due to

1. the national nature of the digitalisation process in nursing and
2. the different educational standards of nurses compared to international standards, which in turn influences the scope of practice.

The digitalisation process is determined by many factors e.g. existing structures and offerings of the healthcare system, the support of care processes, cultural aspects, national political efforts and legislation. A national perspective on this issue is also justified by the previous educational structures in Germany for nurses and the resulting skill and grade mix for the organisations. Most nurses are not academically trained, but have completed three years of training in vocational schools and nursing schools [19]. About 2-3.2% of nurses have an academic qualification, most of them on a bachelor’s level [20]. 

The aim of the DiFuSiN (Digital Future Skills in Nursing) project was to develop a module for a Bachelor degree programme in which the students know and understand the significance, challenges and fields of activity of digital transformation in the profession and learn how to deal with them in a way that is self-designing. In order to strengthen the scientific basis for the development of the module, experts were consulted to identify important core skills. The research question was: what skills need to be addressed in bachelor nursing students in order to meet the current and future challenges of digitalisation in healthcare? 

## 2. Methods

Because of the existing research desideratum in this field, qualitative interviews with experts were conducted to answer these questions. Experts were defined as “people with special expertise on the social issues to be researched” [21]. The interviews were conducted to explore this knowledge. Additionally, social situations or processes are to be reconstructed [21]. 

### 2.1. Case selection

Due to the different specificity of the knowledge, a triangulation of the facts is required in order to get all the necessary information. Experts from a range of disciplines were therefore involved. Originally, 30 interviews had been planned but after 24 interviews data saturation was reached so no further participants needed to be recruited. The participants were chosen on the base of their expertise and, in addition, the “mechanism-oriented explanation strategy” was applied [21]. Sampling was carried out successively on the basis of initial analysis results. Consequently, cases were selected to obtain diversity on the subject matter in order to increase the validity of the study. Seven of the experts declined participating due to lack of time. The participants were first contacted via e-mail and then by telephone.

The expert group consisted of a total of 24 people, including experts from the fields of nursing science (n=5), technology manufacturing in healthcare (n=8), nurses (n=4) and pedagogics (total n=5; digital education experts n=3) plus 2 nursing students. It was not possible to recruit any further students. Most of the participants were male (14/24) with a mean age of 48 years. The experts in the fields of nursing science and practice had many years of experience in healthcare and nursing as well as in the use of assistive technologies. The experts in technology manufacturing were managers or worked in the higher management of start-up companies that had developed and/or marketed new innovative products in the field of care technologies (technical care aids, software, robots, and monitoring-technologies). 

### 2.2. Data collection

The expert interviews were guided interviews. Since the aim includes the reconstruction, it is useful to ensure that the interviewee provides information on all important aspects by using a list of questions [21]. Another reason for using guided interviews is the fact that the topics to be dealt with in the interviews are determined by the aim of the research and not by the answers of the interviewee. Both criteria support the choice of guided expert interviews as a method of data collection [21]. The guidelines were created according to the Collect-Check-Sort-Subsume-principle [22] and took into consideration the aspects of

1. scope,
2. specificity,
3. depth and
4. personal context [23].

The developed questions have an open character and thus follow the methodological principle of openness. They are meant to explore the interviewees’ knowledge and the meaning they give to this knowledge. Furthermore, the questions were formulated as neutrally as possible in order to avoid influencing the experts’ replies. As the experts differed in their knowledge, the guide was adapted in each case to capture specifics. Also, the guide had questions that were asked of every participant in order to get coherent information [21]. The interview guide was pretested with one of the experts. No changes had to be made. 

As part of the introduction, the participants were informed both in writing and verbally about the goals and procedure of the study, thus forming a relationship between the researcher and the participant. Three of the participants were already known to the researcher. 

Data collection took place either online or by telephone and was digitally audiotaped. One of the interviews was a joint interview with two people, since the participants had requested this. The interviews were carried out by SP and accompanied by AB. Both are females with a Master’s degree in Nursing Science (SP) and eEducation (AB), who were working as researchers at Hochschule Bielefeld – University of Applied Sciences and Arts. Previous assumptions of the research team referred to the fact that different experts have different ideas about relevant topics and skills. In addition, it was unclear to what extent the participants were able to comprehend the students' educational content in general and thus know which qualification or job profile the study programme was aiming for.

Data collection and analysis were conducted between October 2020 and October 2021. The interviews took an average of 50 minutes, repeated interviews did not take place. No field notes were made. The experts were offered a copy of the transcriptions but this was only taken up in a two cases, in which a correction of the transcriptions proved unnecessary. 

### 2.3. Data analysis

The interviews were evaluated using qualitative content analysis, which has the advantage of supporting a systematic, theory-guided procedure [21]. Another argument in favor of using this method is that the investigation object had already been delineated a priori. The analysis was done using a systematic procedure to gain information from the transcriptions, whereby the text was examined for relevant information by means of a coding scheme. The coding scheme was derived inductively from the data [21]. Descriptions were developed for each category to ensure consistent coding of the researchers. Coding was conducted by SP and AB. They conducted joint coding sessions regularly. In particular, difficult-to-code passages with different interpretation possibilities were discussed by the entire research team. MAXQDA software was used for analysis. Due to a lack of time resources, the findings were not checked by the participants. However, the summarized results were discussed with other research groups.

### 2.4. Ethics approval and consent to participate

Informed consent was obtained from all participants. Compliance with the applicable data privacy regulations was ensured by the data privacy officer of Hochschule Bielefeld – University of Applied Sciences and Arts. Ethical review was not required as the professionals were asked to share their opinions and knowledge on a non-sensitive topic. Therefore, the participants were not vulnerable and the previously described aspects to ensure informed consent and data privacy were implemented. The study was conducted in accordance with the recent iteration of the Declaration of Helsinki.

## 3. Results

In total, four main categories were identified. The aim of the skill descriptions was to capture them as concretely as possible, rather than at a theoretical meta-level. Further illustration of the categories can be found in attachment 1 according to the quote numbering. 

### 3.1. Organising successful collaboration with patients, relatives and other professionals (doctors, therapists, IT)

The skills described in this area refer to the cooperation between patients and relatives, between members of the care team and between the IT staff of the institution involved. Including the IT staff of an institution takes into consideration the fact that, under certain circumstances, the technical staff can influence the selection of the technologies, can support their use or contribute to solving problems since they have extensive knowledge of how the technologies work. The following skill should be acquired in this area: If technical interventions are applied in nursing care, the nursing staff are able to reflect on their role regarding the successful use and then implement required measures accordingly. This skill includes the reflection of the nurses’ own role in technology-supported care and the initiation of appropriate interventions to ensure the success of the health technology used (quote 1). For example, in the case of televisits, interventions might include preparing for the consultation to reduce anxiety, identifying key questions, assessing needs and explaining the next situation. During the consultation, nursing interventions could aim to facilitate and maintain communication between the parties involved. This emphasises the role of the nurse as a link between different professional groups and patients and relatives in the context of the use of health technologies. 

Another related skill is the communication of technology-related needs (e.g. fears) and related issues (e.g. expectations of technology, privacy) within the care team and with the institution’s IT staff (quote 2, 3). In this case, it is important to identify the needs of different target groups for assistive technologies, e.g. a sense of control and safety, and to communicate these needs to stakeholders. This skill can be important, for example, if a hospital wants to offer patients an app to help them manage their symptoms. The nurse’s role could be to assist in the selection process by mentioning target specific needs, such as providing the GP with data to support communication and safety.

### 3.2. Supporting patients and relatives in the use of assistive technologies

Here – in contrast to the previous area – the skills focus on working directly with patients and relatives. It includes educational interventions such as training and counselling patients and relatives about assistive technologies (quote 4, 5). This area includes core skills within the module. On the one hand, they are important to compensate for patients’ and relatives’ lack of knowledge about possible technology-assisted interventions, since the lack of information sources makes it difficult to acquire this knowledge on one's own, and even more difficult for certain patient groups, such as people with cognitive difficulties. On the other hand, different non-technological interventions for care problems need to be identified against the background of the needs of the user group (quote 6). To this end, the goals of care, particularly in the case of chronic diseases, and other framework conditions must be taken into account when selecting appropriate interventions. 

They also support patients and families in making shared decisions about health technologies (quote 7). This requires recognising the different perspectives and values of those involved, weighing up the benefits and promoting communication about these issues. Nurses also need to be aware of the legal and ethical aspects of the technology selection process. This includes, for example, data protection regulations and the challenges of using technology with people with cognitive impairment.

Nurses should also be able to support patients and families in the day-to-day use of the selected technologies (quote 8). In particular, the experts considered successful implementation of the technical intervention to be essential for the impact of using the technology to be realised. To support this, it is necessary to understand the interplay between technology, everyday life, disease management and users’ needs, and to know how to integrate new interventions in a sustainable way.

Another skill in the area direct patient care is the evaluation of (automatically) technology-collected patient data and combining or contrasting this with other information (quote 9). Experts believe that more data about patients will become available as technologies such as monitoring systems are increasingly used. To make meaningful use of this data in care, it is necessary to

1. collect and analyse the data and the information it contains, and
2. combine the findings with other, non-quantifiable information about the patient to create a comprehensive picture and initiate appropriate care interventions.

The second step is to communicate this overall picture to the patient and relatives.

### 3.3. Planning and implementing projects related to assistive technologies

Nurses should be able to support digitalisation projects in institutions with their expertise by taking on planning, implementation and reflection tasks (quote 10). This is where the skills described above come into play. In addition, knowledge of existing work processes and resources can be effectively incorporated into implementation processes.

### 3.4. Managing lifelong learning in relation to assistive technologies

As the technology market is constantly changing, it is important to keep up to date with innovations (quote 11). To do this, it is important to be familiar with different information services and to reflect on the information they contain in the context of one's own information needs. This includes reflecting on information about data protection or the impact of health technologies. New media formats in which, for example, patients report on their use and difficulties with assistive technologies can provide a basis for information (quote 12, 13). It is necessary to be able to find and evaluate these new media formats. In this way, these formats can contribute to self-education by increasing one’s own perspective.

## 4. Discussion

Overall, the results illustrate which concrete skills will be expected of nurses in the future. Some of the skills addressed by the experts were not included in the module. In the area of technology production, the experts considered the ability to create use cases, requirements analyses and situation descriptions in which the technology would be used to be helpful. Additionally, taking on leadership roles in collaboration with other care staff was rated as beneficial. These included

1. target group-specific staff training,
2. the collection and analysis of (automatically) technologically collected patient data at institutional level, and
3. the implementation of technological interventions at institutional level.

The research team categorised these skills at Master's level, as additional knowledge in the areas of pedagogy, process monitoring and control, and informatics would be required. At bachelor’s level, students acquire only rudimentary knowledge in this context, as the topics differ from the educational objective. Due to the particular skill mix in nursing in Germany, higher demands are often placed on bachelor graduates, who often take on special roles within institutions, implement projects and take on management functions early in their careers [24], [25], [26]. This trend is quite challenging for graduates, as they have little experience as professionals. The demands made of the experts must also be seen against this background. 

Some of the skills identified in this study have been described in other studies. With regard to the Royal College of Nursing’s concept in the field of digital skills, there are overlaps in the areas of

1. promoting communication in the sense of a professional network,
2. staying up-to-date with evolving technology,
3. providing feedback on technology – articulating benefits and risks, and
4. co-developing digitally enabled ways of working [18].

These aspects are described in a similar way by Bleijenbergh. et al. [17]. Brown et al. identified professional communication with other healthcare stakeholders as a relevant topic in the context of eHealth. They further describe information on the use of digital technologies in the area of patient and family education [5]. Konttilla et al. refer to ethical reflection in vulnerable patient groups [27]. 

In addition to this study, Konttila et al. describe analytical skills in assessing data as an important skill [27]. In the discussion with Konttila et al. it becomes clear that depending on the application of a specific technology, e.g. telenursing, additional organisational skills are required. Wynn et al. similarly discuss skills related to the use of specific technologies [28]. This may be particularly difficult in the context of ever-changing technological developments. It seems more important to discuss basic similarities or typical representatives of a technology group than very specific technologies that are unlikely to be used in future care.

One of the difficulties in referring to existing studies and frameworks in the field of digital skills is the lack of clear definitions of what is meant by digital skills. Different areas such as

1. nursing informatics [29], [30],
2. digital literacy,
3. generic skills such as problem solving, and
4. the use of assistive technologies in general and specific technologies [31] are subsumed and mixed under the term digital skills [32], [33], [34].

This is also due to the fact that some of these aspects are subsumed under the term 'technology'. When this is included as a search term in reviews, all these aspects are covered. In addition, these skills are often described in the context of other healthcare professions without a clear differentiation of nursing skill areas, although they are highly relevant in practice, also against the background of a professional understanding of the role of a nurse [29], [35], [36]. The development of a professional role can only be successful if there is a clear articulation of the specific tasks that nurses undertake in relation to other professions. In addition, in some other studies the skills are described in such a general way that no specific tasks can be derived from them, e.g. “uses eHealth in his or her daily work” or “acts professionally when using eHealth” [17]. The debate highlights the need for further discussion and nursing research in the area of digital skills, exploring and describing skills in detail against a professional understanding of the need for care and the role of nurses. A technology-driven discourse across all health professions is not appropriate as it does not adequately reflect practical care. 

In order to develop a specific module on this basis, it is also necessary to reflect on existing concepts on the basis of the educational objective and existing skills. For example, addressing the effectiveness of technology requires knowledge about the implementation of evidence-based care, which is then transferred to a different context. This then allows reflection on the benefits of a technology. In addition to existing concepts, it is essential to describe skills in such concrete terms, since it can be understood exactly what needs to be done [37]. The scope for interpretation is thus minimized, learning objectives can be derived more precisely and can be addressed and tested in a more targeted manner. Such descriptions thus contribute to a design of teaching that is appropriate for the target group [38].

The study has several limitations. The students’ perspective might be underrepresented, since only a few were interviewed. However, their statements coincide with the assessment of the other participants. Another limitation of the findings might be the fact that only experts from Germany were interviewed. This occurred because it was assumed that they would be able to understand the students’ goals, could contribute to current political and ethical debates and take cultural and acceptance-related aspects into account.

## 5. Conclusion

Overall, the experts describe a wide range of specific skills that future bachelor students would need to acquire in order to successfully use technological interventions in their everyday work. These skills need to be discussed and developed in the scientific community in the context of a professional understanding of the nursing role and changes in the professional context. In order to address these skills in a targeted manner, concrete teaching concepts are needed that address situation-specific challenges and promote a self-determined approach to them. One example of such an approach is the educational research project DiFuSiN (Digital Future Skills in Nursing) [39]. 

## Funding

This study was funded by the Stifterverband and the Ministry for Culture and Science of North Rhine-Westphalia (214-5.01.03.02 – 146361). The funding bodies had no influence on study design, analysis, interpretation of results and writing this manuscript. Open access was funded by Deutsche Forschungsgemeinschaft (DFG, German Research Foundation) – 490988677 – and Hochschule Bielefeld – University of Applied Sciences and Arts.

## Competing interests

The authors declare that they have no competing interests. 

## Supplementary Material

Quotes to illustrate the categories
