# Supplementary material for: Skills required by bachelor nursing students to meet the challenges of digitalisation in healthcare – results of qualitative expert interviews
Source: GMS J Med Educ. 2025 Jun 16;42(3):Doc34. doi: 10.3205/zma001758 (PMC12286878; doi:10.3205/zma001758)
Supplement: Quotes to illustrate the categories [file JME-42-34-s-001.pdf]

Attachment 1: Quotes to illustrate the categories

The numbering corresponds to the information in the text.

1	“But of course, it’s also a reflection of the entire process because there is a lot of change for the nurses since their role during ward rounds is simply a structuring one, they are the only people present apart from the telepresence robot. The success of the round depends on the nurse being a strong presence in the patient's room, as she is the only professional person physically present. The doctor has only a limited perspective on the patient through the robot. This has to be considered when using this technology and nurses have to be aware of their changed role.” (I11, 17)
2	“But I believe that the nurses of tomorrow will be characterized by a) a very good understanding of the issue of data privacy, i.e. what does it actually mean when I have a patient or resident here who is actually no longer competent? Which of the available systems can I use for this?” (I3, 17)
3	„That I need to raise awareness. That the patients perhaps don’t have much money and can’t simply download the app. Well, come to think of it, we had a project about introducing and initiating a new app somehow and at the end they all asked “Why is this app used by only ten patients?” Yes, because A, they were older. So they didn’t have a mobile phone.” (I7, 61)
4	“(…) that you are in the group of professionals who have first contact with the patient, for example, or with the families in the case of home care, and that you have contact before any social workers or other professionals and you are the first contact person, so to speak. And you can easily pass things on, inform the patients or relatives. And you can also convey certain contents. (…) Well, because I don’t see the doctors there, that they think it is their concern. They will never take on the role of informing patients or relatives, like “Now pay attention somehow to data protection.” Or “This is how you use the app.” Well, that has to be done by another group of professionals.” (I7, 41)
5	“(…) the patients will inquire much more about technical ways of solving problems. For that, advice competence is needed. And yes, it won’t be just therapy questions but also some about, let’s say, digital health too, and the patients will have questions like which app can I use to manage my diabetes. Then we’ll have to be informative, talk about evidences and also say what does something achieve, what does something not achieve. I think that is a challenge that will also come.” (I11, 25).
6	“Well, technology, we must refrain from thinking technology is the center of the world and of whatever initial consideration. Technology is exactly that: one possibility out of many. And in order to be able to perceive this possibility as meaningful and effective in the sense of the care mandate, certain knowledge is required. But, yes, absolutely, it’s about decision-making. What is the best way to reach the goal I have set in the context of the care mandate.” (I13, 11)
7	“But I make decisions together with my clients who are with me. The important indicators for me are to discover how things are in the home and what these people need and then in the end make a joint decision. And these two parties have to be supported in cases where perhaps difficult or complex information from scientific or technical areas has to be made understandable in a translation process so that these people can handle it in this immediate situation and can pick it up quickly.” (I10, 12)
8	“Which role they also have, those who are attending the patient, must be able to answer the patients’ questions regarding the introduction of new technologies because they have to educate the patients with appropriate competence. That will be our task, so they can include this in their self-management, but this is nothing new. That has been the role of nurses up to now, yes, but it is certainly another challenge to fill it with these technical components and competences.” (I11, 35)

9	“Bringing these analytical ideas into nursing. (…) That on the one hand you look more at the data but also retain your own opinion. Well, to work only in a data-driven way and just follow the data is, I believe, never a good thing.” (I1, 39)
10	“Certainly, project management skills are in my opinion a key issue in every form of modern technology because it always involves projects. So, no one implements digital technology tomorrow and says, there it is, hurrah!” (I20, 35)
11	“When you’ve finished your training or studies and are in the everyday working life, 10, 15, 20 years later you’re in a fast-moving age, where things such as (…) VR and Augmented Reality are standard and a part of it. And you should definitely not close your eyes to it and constantly continue your education via adequate portals, even after your training. But I think that is, so to speak, continuous learning, something that should always be part of it.” (I5, 59)
12	“Much more important is how can I incorporate things that are perhaps already on the market meaningfully into patient care. Or maybe how can I myself benefit from this. Now, for instance, looking at team level or even personally, how can I educate myself further using such formats. How can I assess the contents. (…) How can I assess and perhaps evaluate digital contents. That is important, too. Because it is unbelievable how much content there is that has not been well produced. Where the contents have not been reflected on.” (I7, 15ff.)
13	“No, that many more nurses simply inform themselves in other media (…) but are much more open, get information from a podcast, for example, or from a social network, or from, yes, discussions with other groups in these networks. Because that simply enhances and opens up this horizon and you get out of your nursing bubble, or your knowledge bubble. And that simply expands it.” (I9, 41)
